# Lower-mantle iron heterogeneity constrained by the electrical conductivity of Al-bearing bridgmanite

**DOI:** 10.1126/sciadv.aec7875

**Published:** 2026-04-24

**Authors:** Kui Han, Sinan Özaydın, Hongzhan Fei, Lianjie Man, Fei Wang, Artem Chanyshev, Anthony C. Withers, Alexander Grayver, Tomoo Katsura

**Affiliations:** ^1^ Key Laboratory of Earth Exploration and Information Techniques, College of Geophysics, Chengdu University of Technology, Chengdu, China.; ^2^ Bayerisches Geoinstitut, University of Bayreuth, Bayreuth, Germany.; ^3^ School of Geosciences, University of Sydney, Sydney, Australia.; ^4^ School of Earth Sciences, Zhejiang University, Hangzhou, China.; ^5^ Institute of Geochemistry and Petrology, ETH Zürich, Zürich, Switzerland.; ^6^ Institute of Geophysics and Meteorology, University of Cologne, Cologne, Germany.; ^7^ Center for High Pressure Science and Technology Advanced Research, Beijing, China.

## Abstract

Iron distribution in Earth’s lower mantle profoundly influences planetary evolution by regulating mineral density and mantle dynamics but remains poorly constrained due to trade-offs between temperature and composition in seismic interpretations. Here, we resolve this challenge by measuring electrical conductivity of Al-, Fe-bearing bridgmanite, the dominant lower-mantle mineral, as a function of iron content (*X*_Fe_) under conditions reaching 2000 K and 27 GPa. Bridgmanite conductivity increases dramatically with *X*_Fe_ following an *X*_Fe_^3.6^ power law, while showing minimal temperature dependence. This pronounced sensitivity enables direct inference of global iron variation from geomagnetic-derived conductivity models. Our analysis reveals iron enrichment in large low–shear velocity provinces, supporting their thermochemical rather than purely thermal nature. We identify extensive high-iron regions beneath the western Pacific extending below 1000 km, indicating a vast basaltic reservoir of subducted oceanic crust. These findings provide independent constraints on Earth’s chemical composition and evolution.

## Teaser

The Al, Fe-bridgmanite electrical conductivity is more sensitive to iron content than temperature in the lower mantle.

## INTRODUCTION

Iron (Fe) is the fourth most abundant element in the Earth’s mantle (*1*). Its incorporation into silicate minerals not only affects their density (*2*), thereby influencing mantle convection (*3*), but also regulates the redox conditions of the Earth’s interior due to iron’s variable oxidation states (metallic, ferrous, and ferric) (*4*). These redox changes may have played a key role in the Great Oxygenation Event (*5*), promoting planetary habitability (*6*). Understanding the iron content and distributions in the Earth’s mantle is thus critical for investigating the dynamics and evolution of Earth (*3*, *7*).

Despite its importance, the iron content and distribution within the Earth’s interior, particularly in the lower mantle, remain poorly constrained, due to the scarcity of natural samples (*8*). Although the pyrolytic mantle model suggests a bulk Fe/(Mg + Fe) ratio of 0.1 in bridgmanite, the dominant mineral comprising 80 vol % of the lower mantle (*9*), substantial compositional heterogeneity is expected from geophysical constraints (*10*) and is evident in natural bridgmanite samples (*X*_Fe_ = 0.04 to 0.13) and mineralogical variations ranging from *X*_Fe_ = 0.07 in harzburgite to *X*_Fe_ = 0.37 in basalt (*11*, *12*). However, seismic models have been facing challenges in distinguishing the iron content from the temperature effect due to a trade-off in their effects on seismic velocity (*10*). In contrast, the electrical conductivity of mantle minerals is highly sensitive to iron and H_2_O content (*13*, *14*). Given the limited H_2_O solubility in bridgmanite (*15*), its electrical conductivity is likely dominated by iron content (*16*, *17*). Consequently, comparing the experimentally determined iron-content dependence of bridgmanite’s electrical conductivity with the global three-dimensional (3D) conductivity models offers an opportunity for constraining iron distribution in the lower mantle (*18*, *19*).

Most of the previous experimental studies on bridgmanite conductivity under lower-mantle conditions focused on pressure and temperature dependence (*16*, *20*–*22*), whereas the Fe-content dependence was poorly constrained (*17*). Although iron-content dependence has been investigated in conductivity studies of both bridgmanite-ferropericlase assemblages and bridgmanite, these experiments were limited to temperatures up to 673 K (*16*, *23*), substantially below the ~2000 K conditions at the top of the lower mantle (*24*). Because the conduction mechanism of bridgmanite might be different between 673 and 2000 K (*21*, *25*), the quantitative relationship between iron content and conductivity of bridgmanite under lower-mantle conditions remains unknown.

Here, we report the electrical conductivity of Al-, Fe-bearing bridgmanite as a function of iron content (*X*_Fe_) from 0.10 to 0.37 in per formula unit (pfu) over a temperature range from 400 to 2000 K, using a multi-anvil apparatus and impedance spectroscopy (table S2). These results allow us to construct 1D conductivity profiles for different compositional models of the lower mantle and to constrain the spatial variation of *X*_Fe_ by comparison with 3D global conductivity structures inverted from geomagnetic deep sounding data (*18*).

## RESULTS

### Temperature and *X*_Fe_ dependences of bridgmanite electrical conductivity

**Fig. 1.**
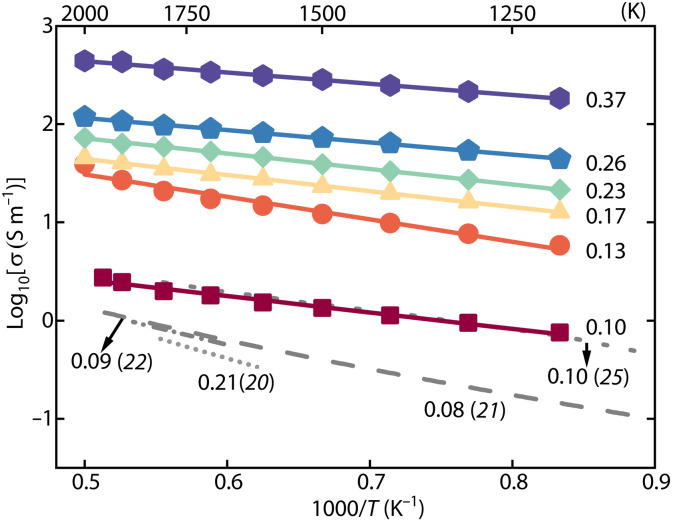
Electrical conductivity of Al-, Fe-bearing bridgmanite with various iron content as a function of reciprocal temperature. The solid lines represent the best-fit curves for the conductivity data, corresponding to the mole fraction of Fe (pfu). The gray lines are Al-, Fe-bearing bridgmanite with varying *X*_Fe_ from previous studies. The uncertainty of conductivity data is smaller than the symbol size.

**Fig. 2.**
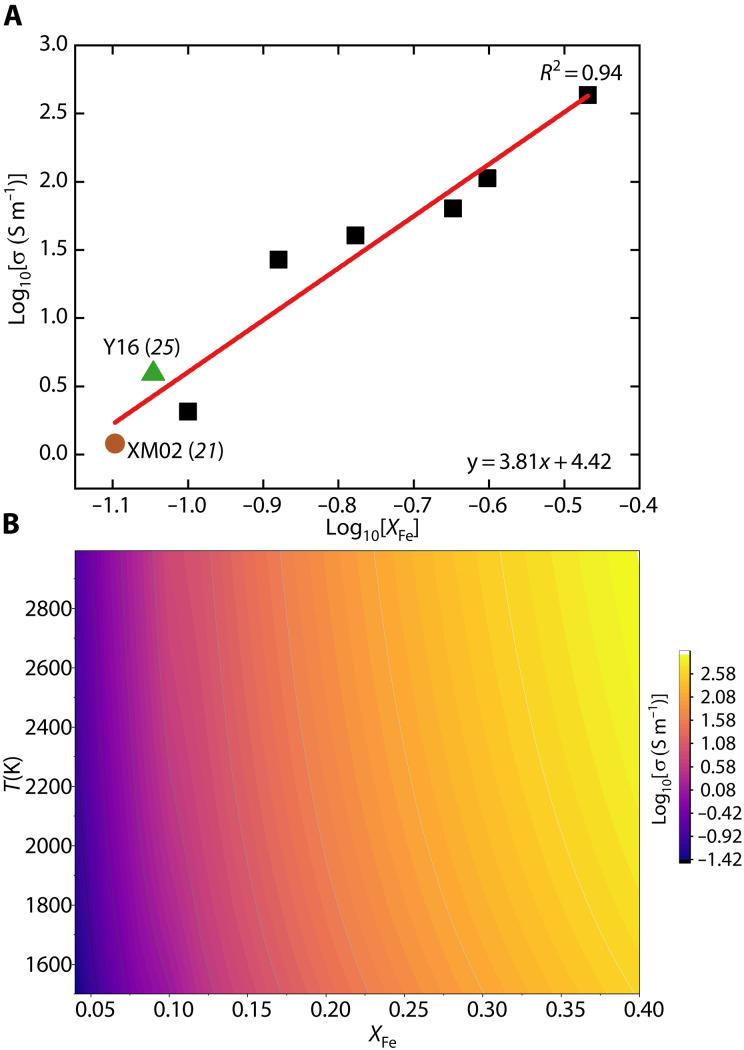
The correlation between electrical conductivity of Al-bridgmanite and mole fraction of iron content (*X*_Fe_) and temperature. (**A**) The logarithmic electrical conductivity as a function of logarithmic *X*_Fe_ at 1900 K. The green triangle and dark orange circle are bridgmanite from the previous studies of Y16: Yoshino *et al.* (*25*); XM02: Xu and McCammon (*21*). (**B**) The comparison of temperature and *X*_Fe_ dependence of conductivity.

The experimental results show that the electrical conductivity of bridgmanite increases systematically with increasing temperature (Fig. 1) and *X*_Fe_ (Fig. 2). The temperature dependence is correlated with *X*_Fe_, i.e., the activation enthalpy of electrical conductivity is a function of *X*_Fe_ (fig. S5). The data were fitted to an empirical form of the Arrhenius equation that incorporates the compositional dependence (*13*)

$$\sigma =\sigma_{0}\cdot X_{\text{Fe}}^{\alpha }\cdot e^{\left(-\frac{H_{a}^{0}-\beta X_{\text{Fe}}^{1/3}}{\mathrm{kT}}\right)}$$ (1)

where σ is the electrical conductivity, σ_0_ is a preexponential factor, α is the exponential factor of *X*_Fe_, *H*_a_^0^ is the activation enthalpy for *X*_Fe_ = 0, β is a constant accounting for geometrical factors, *k* is the Boltzmann constant, and *T* is the absolute temperature. Least squares fitting gives σ_0_ = 10^4.8±4.6^ S/m, α = 3.6 ± 0.3, *H*_a_^0^ = 0.48 ± 0.14 eV, and β = 0.3 ± 0.2. The experimental and analytical details are given in Materials and Methods.

The relatively low activation enthalpy (the term $H_{a}^{0}-\beta X_{\text{Fe}}^{1/3}$ in Eq. 1) and high α documented in our experiments indicate that bridgmanite electrical conductivity is more sensitive to *X*_Fe_ than to temperature (Fig. 2B). At *X*_Fe_ = 0.12, our model shows that a 500 K temperature increase (from 2000 to 2500 K) enhances conductivity by only 0.16 log units, and this enhancement decreases with increasing *X*_Fe_. Given that lateral temperature variations at a given depth are typically less than 500 K (*26*) and that the adiabatic temperature ranges from 1960 K at 660 km to 2580 K at 2800 km in the lower mantle (*24*), the resulting conductivity variation due to temperature should be generally less than 0.2 log units. In contrast, increasing *X*_Fe_ from 0.04 to 0.14 at 2000 K increases conductivity by more than 2 log units, demonstrating that iron content exerts a considerably stronger influence than temperature. Considering the large potential range of iron content of bridgmanite (*X*_Fe_ = 0.04 to 0.37) in the lower mantle (*8*, *12*), we suggest that variations in *X*_Fe_ overwhelmingly dominate the bridgmanite conductivity at a given depth. By comparison, temperature plays only a minor role in controlling bridgmanite conductivity throughout the lower mantle.

Our bridgmanite conductivity at *X*_Fe_ = 0.10 agrees with previous studies at the same *X*_Fe_ (0.10) (Fig. 1) (*25*). The strong *X*_Fe_ dependence explains the higher conductivity observed in our study compared to previous studies at lower *X*_Fe_ (0.08 and 0.09) (*21*, *22*), where *X*_Fe_ effects are particularly pronounced. The lower conductivity reported for higher *X*_Fe_ (0.21) may be influenced by several experimental factors (*20*), as discussed in that study, including the reported uncertainty of up to ~50% in sample thickness. In addition, possible Fe migration into Pt electrodes and contributions from the sample regions beneath the electrodes, both of which are difficult to resolve by x-ray imaging, could potentially affect the measured conductivity (*20*). Although these factors cannot be fully quantified here, they may partly account for the observed differences.

### Conduction mechanism

The nearly linear relationship between the reciprocal temperature and logarithmic conductivity in Arrhenius plots indicates an unchanged conduction mechanism throughout the entire studied temperature range (Fig. 1 and fig. S4). The strong *X*_Fe_ dependence of conductivity, together with the low activation enthalpy (0.26 to 0.47 eV) indicates small polaron conduction as the dominant mechanism controlled by electron hopping between Fe^2+^ and Fe^3+^ (*23*, *27*). Water-controlled proton conduction, which is frequently observed in upper-mantle minerals, such as olivine (*14*), wadsleyite and ringwoodite (*28*), can be excluded because of the absence of H_2_O in our bridgmanite (fig. S2). It was previously thought that the conduction mechanism shifts from small polaron to ionic conduction at higher temperatures (>1600 K) based on deviations from a linear relationship (*21*, *25*). However, our results show no such deviations across the temperature range from 400 to 2000 K. The deviations might be attributed to the high concentration of oxygen vacancies associated with Fe^3+^ in the Si site and the incorporation of Na^+^ (*25*).

The Fe^3+^/ΣFe ratio may also influence bridgmanite conductivity. Large differences in Fe^3+^/ΣFe between Al-free and Al-bearing bridgmanite can affect conductivity by approximately half an order of magnitude (*22*). However, among Al-bearing samples with Fe^3+^/ΣFe ratios of 0.60 to 0.80, no systematic conductivity variation was observed (*25*). Given the comparable Fe^3+^/ΣFe ratios and similar Al content among our samples (table S2), we consider the influence of Fe^2+^/Fe^3+^ to be secondary compared to that of the *X*_Fe_. A large oxygen fugacity variation in the actual lower mantle may nevertheless produce a wider range of Fe^3+^/ΣFe ratios, potentially leading to greater variability in conductivity than predicted by a simple *X*_Fe_-controlled model.

Although the polaron model predicts conductivity proportional to *X*_Fe_^2^ (*29*), our study shows proportionality to the 3.6th power of *X*_Fe_. Increasing *X*_Fe_ not only increases the concentration of small polaron but also reduces the distance between Fe^3+^ and Fe^2+^, thereby facilitating electron- or hole-hopping (*13*, *30*). The *X*_Fe_ dependence could be affected by multiple factors, such as crystal structure (*30*) and defect complexes (*29*). Our *X*_Fe_ dependence (α = 3.6) is similar to that reported for the bridgmanite-ferropericlase assemblage (α = 3.56 ± 1.32) (*16*) but is lower than that of Al-free bridgmanite (α = 5.5) (*23*), which is likely attributable to the narrower *X*_Fe_ range (0.56 to 0.77) investigated in their samples. The fundamental mechanism governing *X*_Fe_ dependence remains unresolved (*29*), necessitating further systematic experimental and theoretical investigations.

## DISCUSSION

### One-dimensional electrical conductivity profile in the lower mantle

The average composition of the whole mantle is assumed to be pyrolytic. However, subduction of oceanic lithosphere introduces basalt and harzburgite into the lower mantle, creating compositional heterogeneity. The electrical conductivity of the lower mantle reflects the major mineral assemblage in these components, dominated by bridgmanite with a subordinate fraction of ferropericlase (*9*). Previous experimental studies showed that at least 30 vol % of ferropericlase is required for the formation of an interconnected framework in the bridgmanite-ferropericlase system (*31*). Because the fractions of ferropericlase in the pyrolite and harzburgite compositions are only 15 and 22 vol %, respectively (*9*), its contribution to the bulk conductivity is minor. Accordingly, we argue that bridgmanite exerts primary control on the lower-mantle conductivity.

With increasing depth in the lower mantle, electrical conductivity in melt-free regions is primarily governed by radial variations in pressure, temperature, and iron content (*X*_Fe_), assuming similar aluminum content. The strong dependence of bridgmanite conductivity on *X*_Fe_ implies that lower-mantle conductivity correlates closely with iron variation. The *X*_Fe_ in bridgmanite decreases gradually with increasing depth due to a reduction in the Fe-Mg partition coefficient (*K*_D_) between ferropericlase and bridgmanite (*32*). Temperature plays a secondary role in the lower mantle, as variations along the adiabatic geotherm (~620 K) result in measurable but relatively minor changes in conductivity (*24*), as discussed above. We consider the effect of Fe^3+^/ΣFe ratio in bridgmanite to be negligible in the upper to middle lower mantle, as this ratio remains ~0.5 to 0.6 up to 50 GPa, covering most of our samples (*4*, *33*). Because the influence of Al is primarily mediated through changes in the Fe^3+^/ΣFe ratio, variations in Al content within a narrow range are expected to have a negligible effect on bridgmanite conductivity (*25*).

Pressure notably influences the electrical conductivity of bridgmanite, with an increase from 25 to 60 GPa resulting in a one order of magnitude enhancement (*20*, *23*, *27*, *34*). The pressure dependence is characterized by activation volume, as described in Eq. 2 in Materials and Methods. Two activation volumes for bridgmanite electrical conductivity have been reported: Δ*V* = −0.55 ± 0.01 cm^3^/mol at *X*_Fe_ = 0.20 (*20*) and Δ*V* = −0.26 ± 0.03 cm^3^/mol at *X*_Fe_ = 0.11 (*27*). As the activation volume varies with *X*_Fe_ (*30*) and *X*_Fe_ = 0.11 approximates the average composition of lower-mantle bridgmanite (*1*, *9*), we adopt Δ*V* = −0.26 cm^3^/mol in this study. Moreover, the conductivity data underpinning the −0.55 cm^3^/mol estimate exhibit relatively large uncertainties (*20*), as discussed before, which further supports the choice of −0.26 cm^3^/mol as the more representative value.

**Fig. 3.**
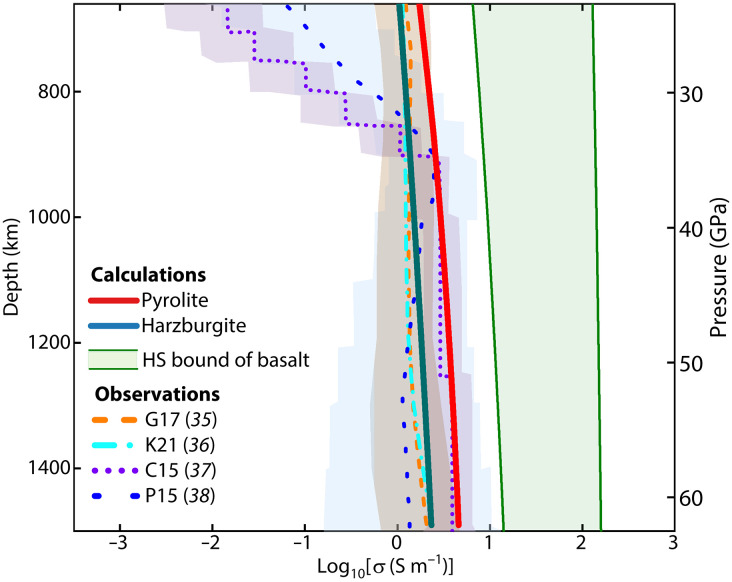
Electrical conductivity profiles of the lower mantle from 660 to 1500 km. The red solid line represents a conductivity profile of pyrolite (*32*), while the dark-green solid line is the conductivity profile of harzburgite with an assumed *X*_Fe_ = 0.07 (*11*). The conductivity of basalt is constrained by the Hashin-Shtrikman (HS) bounds, as detailed in Materials and Methods. The dashed and broken lines are 1D conductivity profiles based on geophysical observations, with shaded areas of corresponding colors indicating their associated uncertainties. An adiabatic geotherm was used to determine the temperature of the lower mantle (*24*).

We have calculated electrical conductivity profiles of pyrolite, basalt, and harzburgite as a function of depth for the upper to middle lower mantle (660 to 1500 km), integrating our bridgmanite conductivity model (Eq. 1) and pressure dependence (see Eq. 2 in Materials and Methods). The conductivity of pyrolite is 1.7 to 4.5 S/m at 660- to 1500-km depths (Fig. 3), which agrees with the 1D global average of conductivity profiles derived from ground and satellite geomagnetic observations, within their reported uncertainties (*35*–*39*). In harzburgite, the bridgmanite *X*_Fe_ is lower than that in pyrolite (*11*), resulting in reduced lower mantle conductivity. Basalt has a notably higher conductivity than pyrolite and all the observed profiles due to the high conductivity of iron-rich bridgmanite (*X*_Fe_ = 0.37) (*12*). This suggests that the lower-mantle composition at depths of 660 to 1500 km is more likely to be pyrolytic than harzburgitic and basaltic.

### Iron content distribution in the lower mantle

**Fig. 4.**
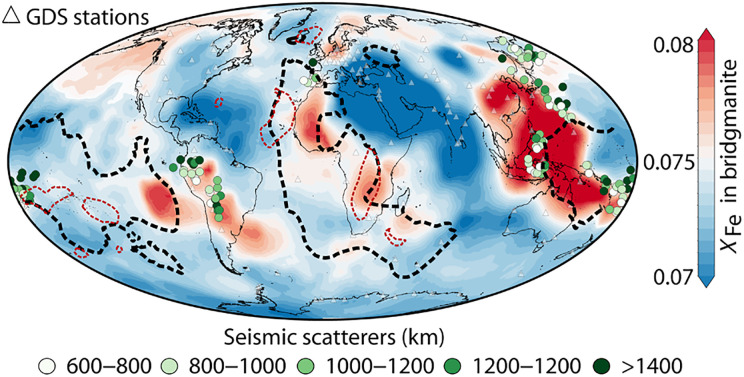
Global *X*_Fe_ distribution derived from the global conductivity model at 1225 km. The seismic scatters were observed in varying depths of the lower mantle (*50*). The red and black dash lines represent the LLSVPs extended at 1200 and 2700 km, respectively, adopted from the vote maps of tomographic models (*42*).

Leveraging the established correlation between bridgmanite electrical conductivity and *X*_Fe_, we calculated the *X*_Fe_ distribution in the lower mantle from a high-resolution global electrical conductivity model (2° × 2°) that uses geomagnetic depth sounding (GDS) measurements (fig. S6) (*18*). The inversion for the *X*_Fe_ is carried out with the pide Python library (*40*), using the same pyrolytic composition, conductivity models, and phase-mixing relationship as the 1D conductivity profile calculations. In the uppermost lower mantle (670 to 900 km), electrical conductivity varies with interspersed high- and low-conductivity zones, lacking distinct regional patterns. On the other hand, at deeper mid-mantle depths (1200 to 1600 km), high-conductivity regions cluster beneath Pacific subduction zones, whereas low-conductivity areas were observed under the Mediterranean-Arabian regions (*18*, *41*). Because geomagnetic observations constrain only relative spatial variations in conductivity, the inferred *X*_Fe_ distribution should be interpreted as relative patterns rather than absolute values (*18*, *41*). Under the corresponding pressure-temperature conditions (*24*), we present *X*_Fe_ distribution at representative depths of 825 km (fig. S7) and 1225 km to illustrate these spatial variations (Fig. 4).

A notable feature at 1225 km is the high *X*_Fe_ beneath Northwest to Southern Africa, roughly coincident with the vertical extension of large low–shear wave velocity province (LLSVP) that extends to a depth of ~1200 km (Fig. 4) (*42*). LLSVPs are characterized by low shear wave velocities (*7*) and are estimated to have higher density than the ambient mantle due to their long stability at the base of the mantle (*43*), indicating a thermochemical origin (*3*, *6*, *44*). The denser composition of the LLSVP, attributed to Fe enrichment, is further corroborated by the spatial alignment with geoid highs under Africa (*45*, *46*). While a second LLSVP exists beneath the Pacific Ocean (*7*), it extends beyond the reliable depth resolution of geomagnetic induction studies (<1600 km) (*18*).

Another prominent feature is the high-*X*_Fe_ regions at 1225-km depth beneath the western Pacific and South America. Because they show proximity to major subduction zones, we propose that these high-*X*_Fe_ regions represent subducted iron-rich basaltic material that has penetrated the lower mantle (*11*, *47*). While some basaltic crust may segregate and accumulate near the 660-km discontinuity during slab subduction (*48*, *49*), another fraction descends to the deep lower mantle, forming seismic scatterers (*50*). The spatial correlation between these seismic scatterers and our iron-rich regions supports our hypothesis that these regions may indeed be relics of subducted basaltic material in the mantle. As these scatterers are detectable only during the post–stishovite phase transition, whose depth varies with Al_2_O_3_ and water content (*51*), they likely reflect only a portion of the total basalt present. This inherent observational bias suggests that the actual basalt distribution is likely more extensive (*49*, *52*), fully aligning with the widespread high-*X*_Fe_ regions we interpreted at 1225 km (Fig. 4). Furthermore, the spatial correlation between these high-*X*_Fe_ regions and geoid highs in the western Pacific and South America provides further support for the presence of extensive, dense basaltic materials in the lower mantle (*45*). Forthcoming high-resolution studies focusing on morphology of this basalt reservoir could elucidate its role in mantle convection dynamics.

As described in Materials and Methods, the *X*_Fe_ variation pattern may vary for different conductivity models, particularly when comparing models that are based on ground-based observatories or those that also used satellite data, which differ significantly in their methodologies, spatial coverage, and resolution. Our comparison with the *X*_Fe_ variations derived from the satellite-based conductivity model at 1225 km (fig. S8) reveals broad consistencies as well as some differences between models (*36*). The high-*X*_Fe_ signatures beneath major subduction zones (western Pacific and South America) and the Southern African LLSVP are robust features present in both models, strengthening the interpretations presented earlier in the discussion. However, differences in regions such as North America and northwestern Africa underscore the need for improved constraints on lower-mantle conductivity structure (*53*). The consistency between these two independently derived models supports our primary conclusions. Nevertheless, systematic investigations into the effects of different inversion methodologies, including regularization strategies, source parameterizations, and the treatment of ionospheric versus magnetospheric signals, are essential to reconcile these model differences and improve confidence in lower-mantle conductivity interpretations (*53*, *54*).

## MATERIALS AND METHODS

### Synthesis of bridgmanite

MgO, SiO_2_, FeO, Fe_2_O_3_, and Al_2_O_3_ powders from Sigma-Aldrich were used as starting materials. The FeO and Fe_2_O_3_ were dried at 500 K, whereas the others were dried at 1273 K over 2 hours before weighing. The Fe_2_O_3_ was enriched with 20 wt % of ^57^Fe for Mossbauer spectroscopy analysis. Mixtures with bulk compositions following table S1 were prepared by grinding in ethanol. After drying in an oven at 120°C for more than 24 hours, the mixtures were fused at 1923 K for 2 hours, followed by a rapid quench in cold water. Last, homogeneous glass was obtained as confirmed by scanning electron microscopy (SEM). One glass sample was ground into a powder and heated in a gas-mixing furnace at 1523 K for 48 hours with an oxygen fugacity of 2 log units below the fayalite-magnetite-quartz buffer (or near IW) in a CO_2_-CO gas-mixing furnace to reduce Fe^3+^ to Fe^2+^.

The glass and reduced powder were filled into rhenium capsules and fitted into 7/3 cell assemblies with LaCrO_3_ furnaces. Each cell assembly was then compressed to 24 or 27 GPa and heated at 1973 K for 4 hours (one run at 2373 K for 100 min). Last, single-phase bridgmanite samples were synthesized, as confirmed by x-ray diffraction and SEM (fig. S2). Small amounts (<2%) of stishovite appeared in run I1696, which will not affect the electrical conductivity measurements because of the lack of interconnection between stishovite grains. All the synthesized samples were cut into discs with a 0.2/0.3-mm thickness and a 0.5-mm diameter for electrical conductivity measurements.

### Electrical conductivity determination

The bridgmanite samples were sandwiched between two Mo or Re electrodes within an Al_2_O_3_ sleeve and insulated from the Re heater by an MgO sleeve (fig. S1). A set of thermocouples (WRe_3_-WRe_25_) was connected to one of the Mo/Re electrodes for measuring the sample temperature, while an additional W-Re wire was connected to the other Mo/Re electrode. The current “Gen output” and voltage “HI” terminals of the Solartron 1260 are connected through two thermocouple wires to one sample electrode, while a third W97Re3 wire connects the opposite electrode to the current “Input” and voltage “LO” terminals. All wires are placed between tungsten carbide cubes except those connected to the heater, ensuring that the heater circuit does not influence the measurement. Under this configuration, only a single W97Re3 wire contributes to the series resistance. To minimize this contribution, we used a very short wire segment whose dc resistance was measured to be ~2.7 Ω at room temperature. Furthermore, in the outer part of the pressure medium and the gasket, the electrode wire was enclosed within a Cu coil, which provides mechanical and thermal support. Because Cu has much lower resistivity and forms a parallel conduction path, the effective resistance of the electrode assembly is even smaller than 2.7 Ω. Therefore, subtracting 2.7 Ω from the real part of the impedance represents a conservative correction, and the wire resistance is negligible compared with the sample resistance at all temperatures.

A Cr_2_O_3_-doped MgO octahedron with 7-mm edge lengths was used as a pressure medium. A thermally insulating ZrO_2_ sleeve was placed outside the heater. Prior to assembly, the pressure medium and all the ceramic parts, including MgO, Al_2_O_3_, and ZrO_2_, were dried at 1273 K for more than 2 hours. No glue or cement was used in the assembly to avoid any influence of volatiles on conductivity measurements. The background resistance of the assembly was determined by replacing the bridgmanite sample with a dense Al_2_O_3_ disc, whose conductivity was more than two orders of magnitude lower than that of the sample (fig. S4). This verification is particularly important under high pressure, where the small size of the assembly components could lead to reduced insulation resistance, potential current leakage, or partial shorting through anvil-heater-anvil pathways. The background test is therefore essential to ensure that the measured impedance originates solely from the sample.

Each assembly was compressed to 27 GPa using tungsten carbide cubes with 3-mm truncation edge, followed by impedance spectroscopy analysis using an AMETEK Solartron 1296 impedance gain-phase analyzer. A 500-mV ac voltage with a frequency ranging from 0.1 to 10^7^ Hz was used. Impedance analysis was performed during heating-cooling cycles with a temperature interval of 50 or 100 K. More than one heating-cooling cycle was performed for each sample to ensure reproducibility. At temperatures less than 1800 K, the impedance spectra showed a semicircular arc, which was fitted to an equivalent circuit consisting of a resistor and a capacitor in parallel (fig. S3). The resistance of the sample was derived from the resistor. At higher temperatures, the semicircular arc vanished, while inductance related to the wire leads appeared. The sample resistance was determined from the impedance value at the frequency where the phase shift was zero or close to zero.

After the measurements, the cross sections of the recovered samples show no visible deformation (fig. S2). This indicates that the sample geometry was well preserved throughout the experiment, despite the shear stresses in the 7/3 assembly. The postexperimental thickness of each recovered sample was measured using SEM at ~10 positions. The variability among measurements was <2%, and these uncertainties are now listed in table S2. Because the recovered samples were made into thin sections for spectroscopy analyses, the diameter could not be directly measured after the experiment. Nevertheless, large changes in diameter are unlikely for two reasons: (i) The samples were presynthesized at 24 to 27 GPa, i.e., at pressures equal to or slightly lower than the pressures used during conductivity measurements. Therefore, minimal radial deformation is expected. (ii) Sample thickness changed negligibly before and after experiments, indicating that volumetric strain was also small. To provide a conservative upper bound, we measured the maximum observable diameter in SEM cross sections. This yields an estimated maximum uncertainty of 3.6%. The electrical conductivity (σ) was calculated from the formula σ = *l/S·R*, where *l* is the sample thickness, *S* is the surface area, and *R* is the resistance. Propagating these uncertainties (2% in thickness and 3.6% in diameter) through the conductivity calculation yields a total uncertainty of approximately ±0.03 log units, which does not markedly affect our results or conclusions.

### Sample characterization

The phases of the sample were identified at each stage using a microfocus x-ray diffractometer with a Co anode operated at 40 kV and 500 mA. Backscattered electron and secondary electron images were obtained using an LEO1530 SEM operating at an acceleration voltage of 15 kV. The chemical composition of the samples was determined using a JEOL JXA-8200 electron probe microanalyzer operating at a beam current of 5 nA and an acceleration voltage of 15 kV. The standards for microprobe analysis were corundum for Al, enstatite for Mg and Si, and metallic iron for Fe. The chemical composition of bridgmanite before and after the electrical conductivity measurements is listed in table S1. Electron microprobe analysis shows that Fe was barely lost during the conductivity measurements.

The H_2_O contents of the samples before and after impedance analysis were examined using Fourier transform infrared spectroscopy (FTIR). The bridgmanite samples were double-polished to thin sections with a thickness of 100 to 200 μm. The FTIR analysis was performed using a Bruker Vertex 70 V spectrometer with CaF_2_ beamsplitter and near-infrared light source coupled with a Hyperion 2000 microscope at Bayerisches Geoinstitut. The aperture size varied from 0.6 to 1.5 (corresponding to a beam size of 40 to 100 μm). A total of 128 scans were collected for each specimen at a 4 cm^−1^ resolution. Unpolarized FTIR spectra were collected between 2000 and 10,000 cm^−1^ (fig. S2). More than 10 points were collected for each sample. No hydrogen-related absorptions were observed in the bridgmanite samples before and after conductivity measurements (fig. S2).

The proportion of Fe^3+^ and Fe^2+^ in bridgmanite was determined using Mössbauer spectroscopy conducted in transmission mode with constant acceleration at room temperature. The Mössbauer spectrometer were used with a nominal 370-MBq ^57^Co high specific activity (point) source in a 12-μm Rh matrix. The velocity scale was calibrated relative to α-Fe foil using the positions certified for the standard reference material no. 1541 of the (former) National Bureau of Standards. Line widths of 0.36 mm/s for the outer lines of α-Fe were obtained at room temperature. The effective Mössbauer thickness of the samples varied between 5 and 10 mg Fe/cm^2^. The duration of each measurement varied between 24 hours and 3 days. The obtained spectra were fitted using MossA software with full transmission integral to multiple doublets with pseudo-Voigt line shapes (fig. S2) (*55*). The fractions of Fe^3+^ and Fe^2+^ were determined from the integration of the doublets (*56*). Despite the variation from 0.34 to 0.65 among different samples, the Fe^3+^/∑Fe ratio of each bridgmanite shows negligible change after the conductivity measurement (except for I1779). The derived parameters of the Mössbauer spectra are listed in table S3.

### Electrical conductivity profile and *X*_Fe_ distribution

To calculate the electrical conductivity profiles of pyrolite and harzburgite compositions at depths of 660 to 1500 km, pressure dependence is incorporated into Eq. 1 as

$$\sigma =\sigma_{0}\cdot X_{\text{Fe}}^{\alpha }\cdot \text{exp}\left[-\frac{\left(∆H_{a}^{0}-\beta X_{\text{Fe}}^{1/3}\right)+P\Delta V}{kT}\right]$$ (2)

Here, *P* represents pressure, and Δ*V* denotes the activation volume, with a value of −0.26 cm^3^/mol (*27*). The activation enthalpy, $∆H_{a}^{0}$, was recalibrated by fitting our conductivity data measured at 27 GPa to account for the inclusion of the activation volume. Ferropericlase, assumed to be 20 vol % in both pyrolite and harzburgite (*9*), is included in the bulk conductivity calculation (*57*). The temperature variation with depth between 660 and 1500 km is based on the adiabatic geotherm (*24*). Given that basalt is composed of 32 vol % bridgmanite, 25 vol % davemaoite, 26 vol % calcium ferrite phase (CF-phase), and 18 vol % stishovite (*9*), the electrical conductivities of these constituents are calculated individually based on laboratory-derived conductivity models. The conductivity of bridgmanite (*X*_Fe_ = 0.37) in the basalt composition is derived from the experiments conducted in this study (*12*). For davemaoite, a dry composition is assumed in conductivity calculations (*58*). Stishovite is assumed to incorporate the maximum amount of water (3000 parts per million) (*59*, *60*). Although the CF-phase in basalt has a high *X*_Fe_ (0.26) (*12*), its conductivity has not been previously measured. Therefore, the conductivity of the CF-phase is calculated based on the conductivity model for bridgmanite developed in this study, which may introduce some uncertainty. This approach provides a first-order approximation for basalt conductivity that can be refined when direct measurements of the CF-phase become available. Nevertheless, basalt conductivity remains at least half an order of magnitude higher than pyrolite based on the measurements using diamond anvil cell (*61*). Minor phases such as magnesite, hollandite, and δ-AlOOH, potentially introduced by metamorphism during subduction, are unlikely to substantially affect the bulk conductivity due to their limited volume fraction (*9*). The Hashin-Shtrikman bounds are used to determine the conductivity range of basalt (*62*).

The 670- to 900-km and 1200- to 1600-km layers of the global electrical conductivity model based on GDS were used in the calculation (fig. S6) (*18*). Despite the large discrepancy among the electromagnetic observations in the topmost lower mantle, the conductivity models consistently show high conductivity in the western Pacific subduction zone and low conductivity in the Mediterranean-Arabian region at 1225 km (*18*, *41*, *63*, *64*). We calculated the lateral *X*_Fe_ distribution of bridgmanite at the depths of 825 and 1225 km based on our conductivity measurement with a line-search algorithm using Python library pide (Eq. 1) (*40*). Due to the lack of direct measurements of temperature distribution in the lower mantle, the temperature distribution was calculated using the study that combines phase relations of minerals and seismic data in the mantle transition zone (*26*). We then extrapolate these temperatures to the corresponding lower-mantle depths based on the adiabatic geothermal gradient (*24*). Note that these temperature estimates may be biased due to the varying positions of subducting slabs and mantle plumes relative to the transition zone. Therefore, a global uniform temperature solely based on the adiabatic geotherm was also used to invert the *X*_Fe_ distribution for comparison (fig. S7) (*24*). Although inverted *X*_Fe_ differs slightly between these two temperature models, the relative lateral variation of *X*_Fe_ is less affected, primarily due to the weaker influence of temperature on conductivity relative to iron content.

In addition to the conductivity model adopted here (fig. S6), several mantle conductivity models have been derived from geomagnetic observations using inversion strategies that differ in data sources, spatial parameterization, and regularization schemes (*36*, *41*, *53*, *63*). Ground-based observatory networks provide better resolution in regions with good coverage but have limited sensitivity in oceanic regions; these models yield broadly consistent large-scale conductivity patterns (*18*, *41*, *63*). In contrast, satellite missions, such as CHAMP and Swarm, offer near-global and more homogeneous spatial coverage, although with reduced sensitivity to short-wavelength variations due to challenges in separation of space and time variations (*36*). To assess the robustness of our inferred *X*_Fe_ values with respect to model selection, we performed an independent calculation using the satellite-derived conductivity model based on Swarm data (fig. S8) (*36*). This approach involves two fundamentally different inversion frameworks, ground-based observatories versus satellite-based data, thereby enabling evaluation of the sensitivity of our interpretations to methodological choices, as discussed in the main text.

## Supplementary Materials

### This PDF file includes:

Figs. S1 to S8 Tables S1 to S3 References
