# Supplementary material for: Lower-mantle iron heterogeneity constrained by the electrical conductivity of Al-bearing bridgmanite
Source: Sci Adv. 2026 Apr 24;12(17):eaec7875. doi: 10.1126/sciadv.aec7875 (PMC13108530; doi:10.1126/sciadv.aec7875)
Supplement: Supplementary file 1 — Figs. S1 to S8 Tables S1 to S3 References [file sciadv.aec7875_sm.pdf]

Science Advances
AAAS

Supplementary Materials for

Lower-mantle iron heterogeneity constrained by the electrical conductivity of Al-bearing bridgmanite

Kui Han *et al.*

Corresponding author: Kui Han, hankui@cdut.edu.cn

Sci. Adv. **12**, eaec7875 (2026)
DOI: 10.1126/sciadv.aec7875

This PDF file includes:

Figs. S1 to S8
Tables S1 to S3
References

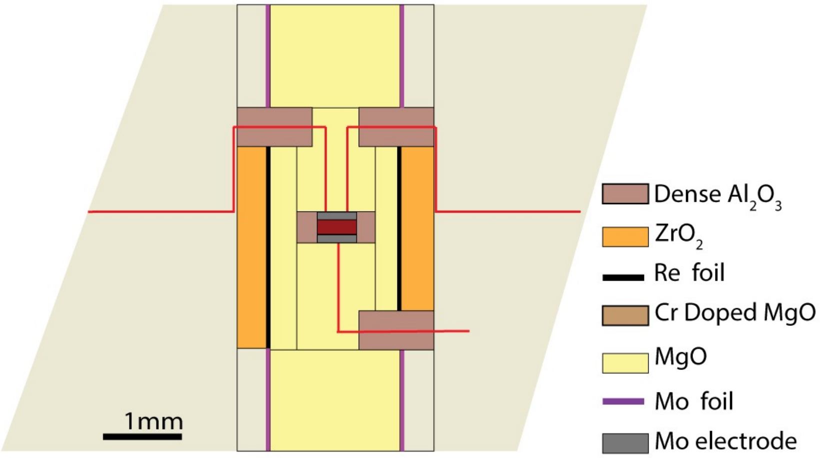

Fig. S1. Assembly cell design. Schematic cross section of the cell (7/3 multi-anvil assembly) for electrical conductivity measurements.

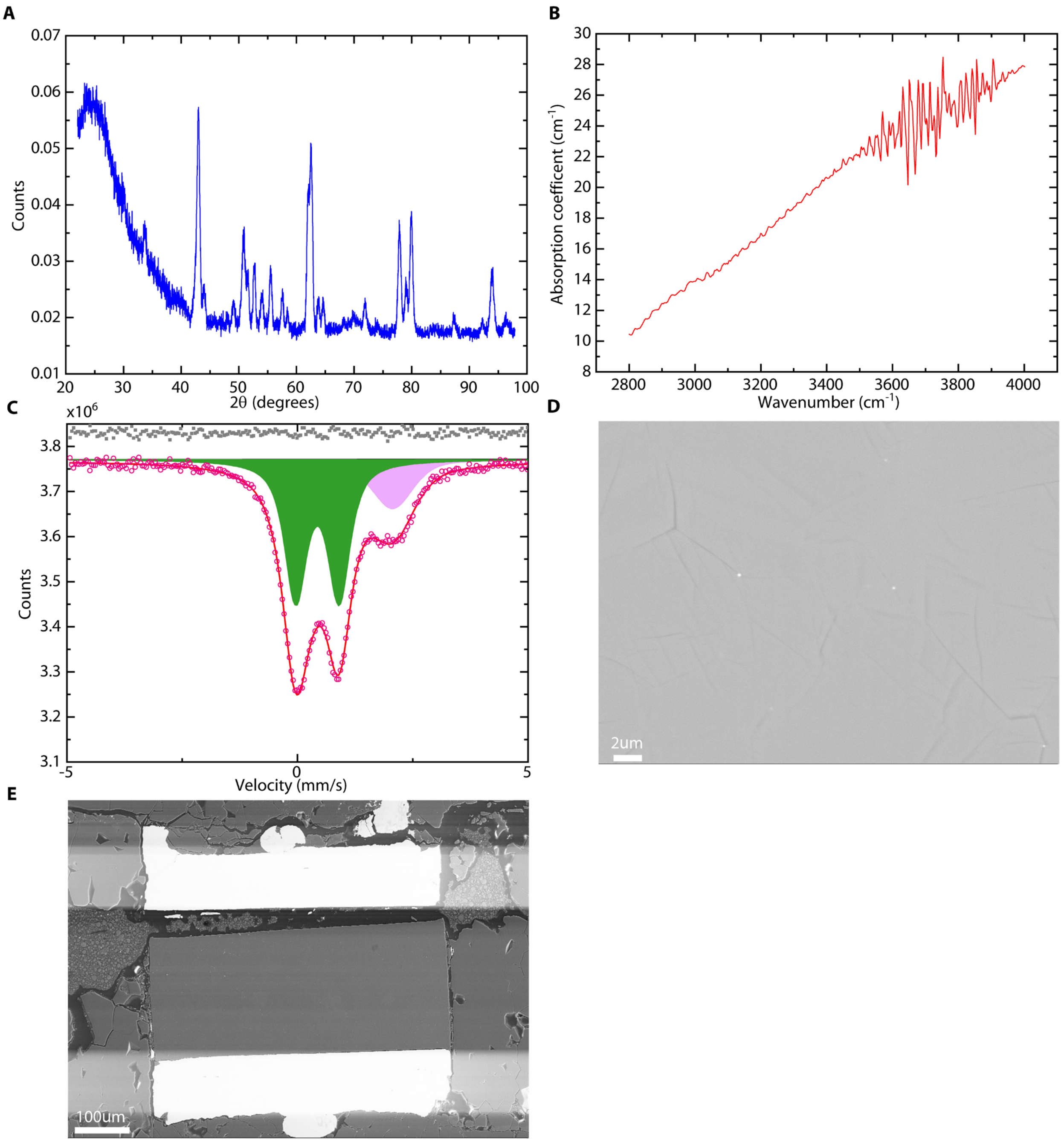

Fig. S2. Representative result for characterization of bridgmanite. X-ray diffraction pattern (**A**), Fourier Transform infrared spectroscopy (**B**), Mössbauer spectroscopy (**C**), Backscattered electron image of I1764 before conductivity measurement (**D**) and the secondary electron image of I1755 cross section after conductivity measurement (**E**).

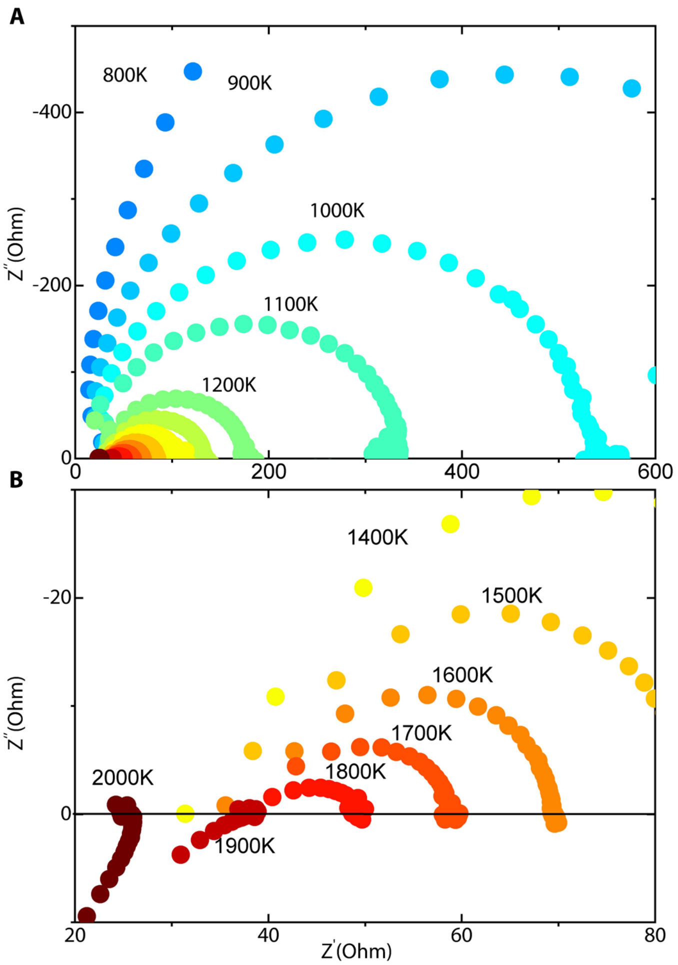

Fig. S3. Representative impedance spectra. The Nyquist plot of the impedance spectroscopy for I1755 at low (**A**) and high (**B**) temperatures, showing the real (X-axis) and imaginary (Y-axis)impedance components.

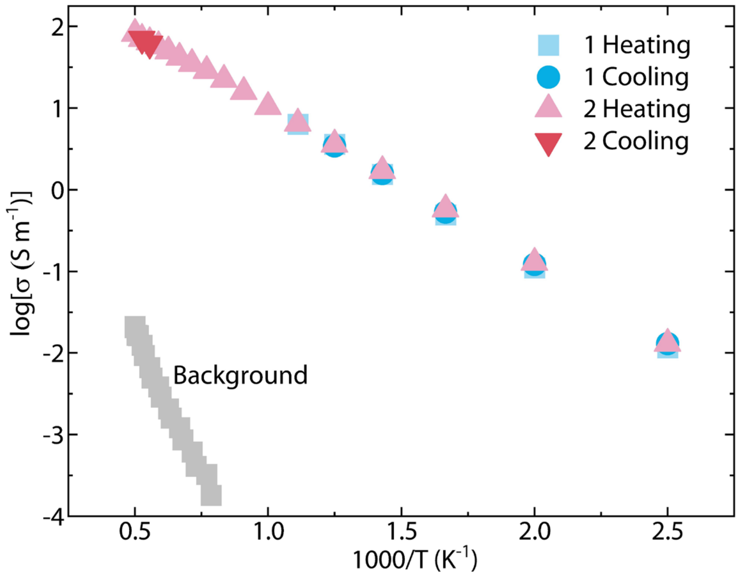

Fig. S4. Representative electrical conductivity -temperature diagram of experimental results. The data shows the logarithmic electrical conductivity of (I1764) as a function of reciprocal temperature at two heating and cooling circles. The background resistance was determined by replacing sample with harden Al_2O_3. The electrical conductivity at the second cooling stage was obtained at two temperature points to ensure reproducibility.

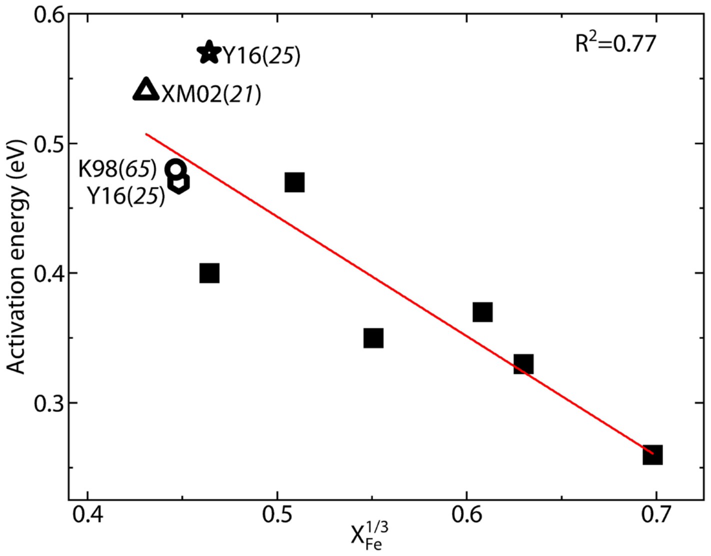

Fig. S5. Relationship between X_{Fe} and activation energy of bridgmanite in small polaron conduction. The solid square represents our data, while the others symbols with X_{Fe} in parentheses derived from previous studies, in which the main conduction mechanism is small polaron (*21*, *25*, *65*).

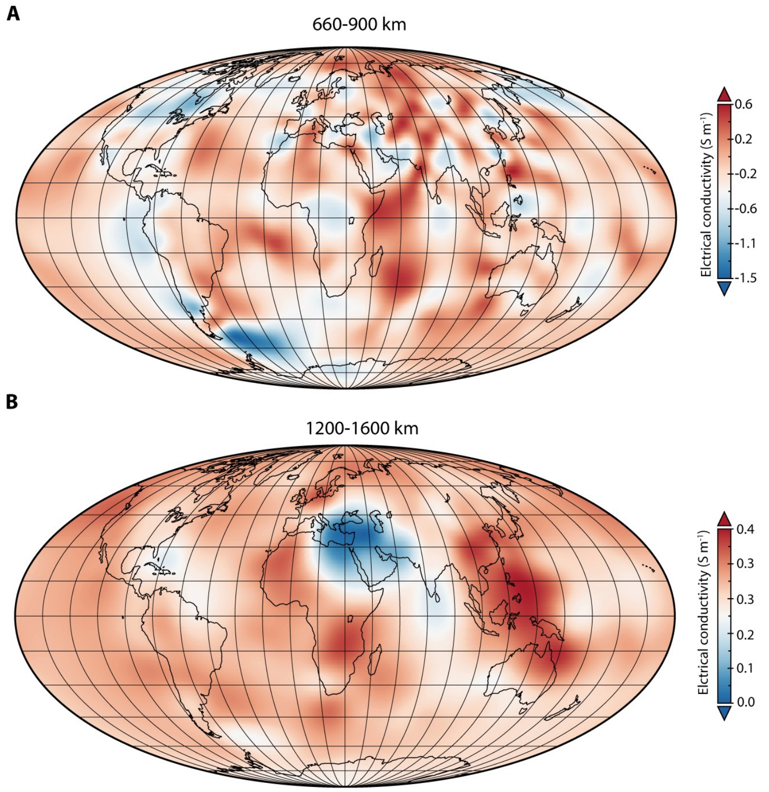

Fig. S6. The global electrical conductivity variation at 670–900 km (A) and 1200–1600 km (B) inverted from geomagnetic induction, adopted from (*18*) .

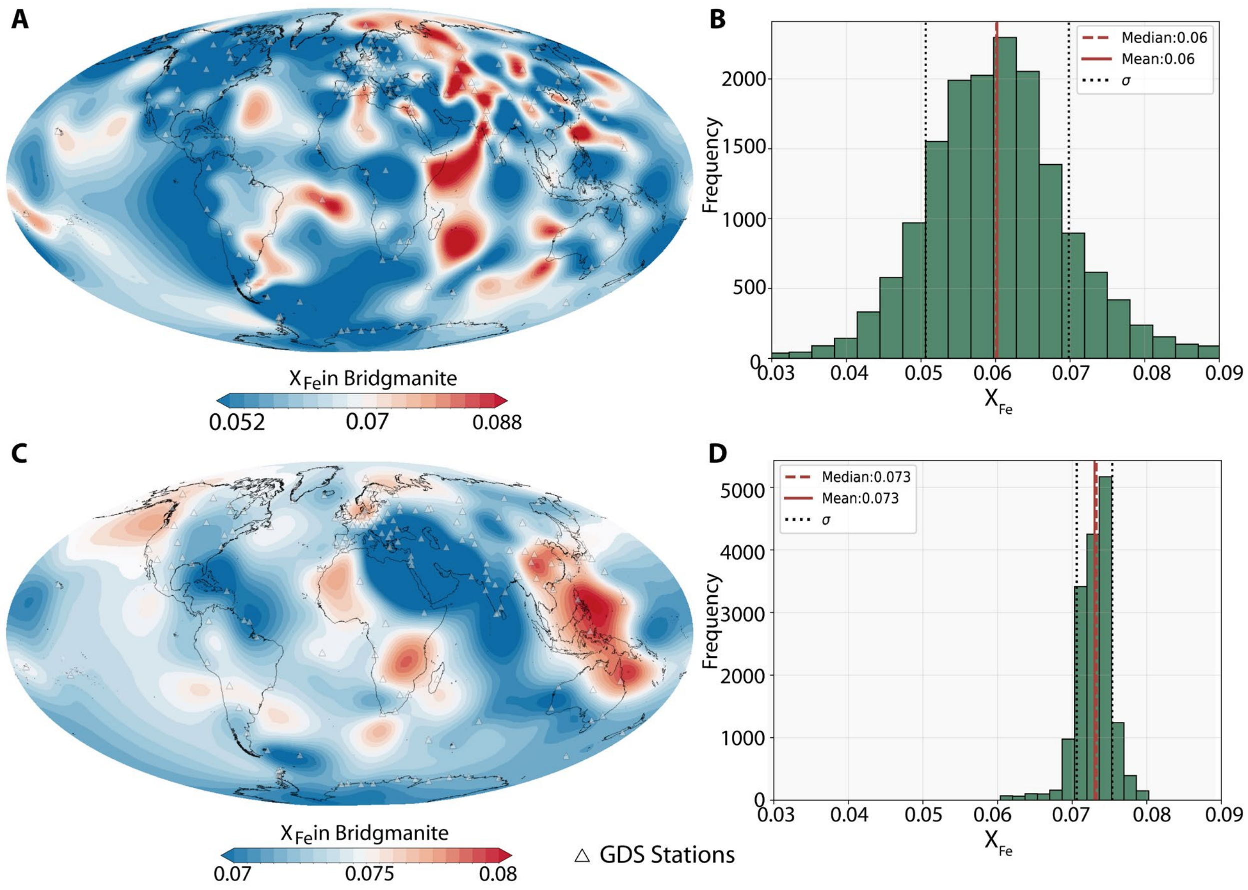

Fig. S7. The global iron content variation without temperature correction at 825 km (A) with its histograms (B) and at 1225 km (C) with its histograms (D). The greater heterogeneity at 825 km compared to 1225 km may reflect both the decreasing resolution of geomagnetic induction with depth and compositional differences across the ~1000 km viscosity discontinuity, where slab stagnation and plume deflection create complex chemical heterogeneity(*66*, *67*).

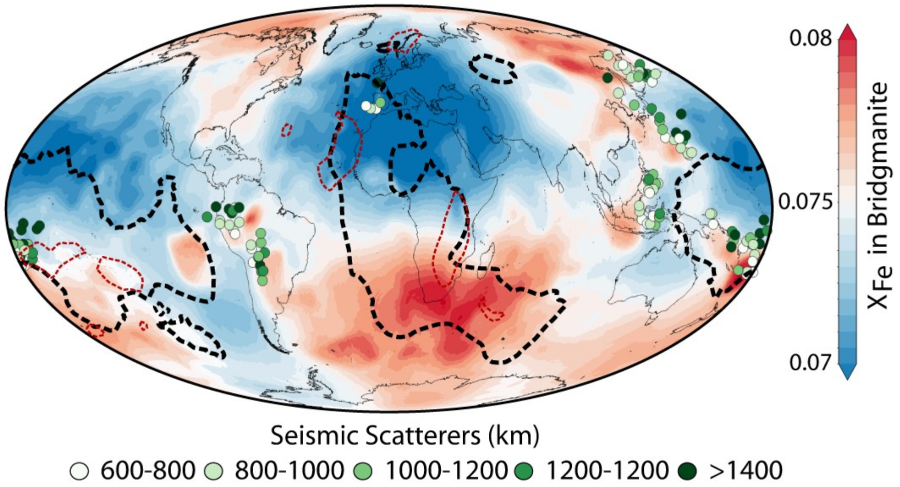

Fig. S8. Global X_{Fe} distribution derived from a satellite-based global conductivity model at 1225 km depth (*36*). The lines are the same as those in Fig. 4.

Table S1. Composition of starting materials and recovered samples

	Sample	SiO2	MgO	FeO	Fe2O3	Al2O3
Starting composition (Mole ratio)	S8059	1	0.9		0.05	0.11
	I1760	0.09	0.82	0.08	0.05	0.05
	I1755	0.9	0.86	0.04	0.05	0.05
	I1764	0.08	0.76	0.04	0.1	0.1
	I1767	0.8	0.72	0.08	0.1	0.1
	I1779	0.72	0.56	0.27	0.05	0.25

		SiO2	Na2O	MgO	CaO	FeO*	Al2O3	Total
Synthesied bridgmanite before conductivity measurement (wt.%)	S8059	53.13	0.05	30.11	0.25	7.31	10.36	101.21
		1.08	0.02	0.44	0.03	0.11	1.42	0.32
	I1760	51.60		31.84	0.25	11.46	5.19	100.35
		0.98		0.83	0.26	0.26	0.14	0.45
	I1755	53.08		32.74	0.34	9.29	5.30	100.86
		0.25		0.29	0.02	0.12	0.09	0.44
	I1764	46.02	0.02	29.01	0.28	14.83	9.78	100.03
		0.36	0.02	0.29	0.03	0.15	0.16	0.58
	I1767	45.17	0.03	27.15	0.27	16.69	9.72	99.23
		1.06	0.02	0.57	0.05	0.33	0.23	0.34
	I1779	41.45	0.05	21.98	0.23	23.40	12.32	99.57
		1.30	0.03	0.63	0.02	0.51	0.33	0.51
Recovered bridgmanite after conductivity measurement (wt.%)	S8059	52.79	0.05	29.91	0.26	7.19	10.40	100.67
		1.37	0.02	1.13	0.08	0.31	1.00	0.57
	I1760	51.22	0.06	31.71	0.27	11.27	5.15	99.91
		0.61	0.03	0.60	0.04	0.30	0.12	0.60
	I1755	52.32	0.06	33.42	0.33	9.06	5.43	100.84
		0.37	0.02	0.46	0.04	0.26	0.15	0.34
	I1764	45.25	0.01	28.73	0.27	14.67	9.69	98.79
		0.34	0.02	0.15	0.02	0.27	0.12	0.40
	I1767	45.57	0.02	27.22	0.25	16.37	9.88	99.53
		0.29	0.02	0.22	0.03	0.25	0.14	0.43
	I1779	42.83	0.04	22.15	0.24	21.60	12.44	99.50
		0.70	0.03	0.58	0.03	0.80	0.21	0.56

*FeO is assumed that all Fe is ferrous iron in the bridgmanite before and after electrical conductivity measurements.
The second rows of the data for each sample correspond to the standard deviation.

Table S2. Summary of experimental runs

No.	P (GPa)	T (K)	X_{Fe} (Formular)	Fe^{2+}:Fe^{3+}	σ_0 (S/m)	Activation energy (eV)	Comments	Thickness (μm)
S8059	27	1950	0.10 ($Fe_{0.10}Mg_{0.77}Al_{0.21}Si_{0.90}O_3$)	63:37			Before EC Exp	
			0.10 ($Fe_{0.10}Mg_{0.76}Al_{0.21}Si_{0.91}O_3$)	66:34	29.6	0.40	After EC Exp	206(3)
I1755	27	2000	0.14 ($Fe_{0.14}Mg_{0.85}Al_{0.11}Si_{0.92}O_3$)	54:46			Before EC Exp	
			0.13 ($Fe_{0.13}Mg_{0.87}Al_{0.11}Si_{0.91}O_3$)	65:35	466.77	0.47	After EC Exp	200(4)
I1760	27	2000	0.17 ($Fe_{0.17}Mg_{0.84}Al_{0.11}Si_{\cdot 91}O_3$)	54:46			Before EC Exp	
			0.17 ($Fe_{0.17}Mg_{0.84}Al_{0.11}Si_{0.91}O_3$)	59:41	378.62	0.35	After EC Exp	201(1)
I1764	27	2000	0.23 ($Fe_{0.225}Mg_{0.78}Al_{0.21}Si_{0.83}O_3$)	36:64			Before EC Exp	
			0.23 ($Fe_{0.23}Mg_{0.79}Al_{0.21}Si_{0.83}O_3$)	35:65	768.42	0.37	After EC Exp	206(1)
I1767	27	2000	0.26 ($Fe_{0.26}Mg_{0.75}Al_{0.21}Si_{0.83}O_3$)	57:43			Before EC Exp	
			0.25 ($Fe_{0.25}Mg_{0.75}Al_{0.21}Si_{0.84}O_3$)	53:47	1167.88	0.33	After EC Exp	317(2)
I1779	27	2000	0.37 ($Fe_{0.37}Mg_{0.63}Al_{0.28}Si_{0.79}O_3$)	36:64			Before EC Exp	
			0.34 ($Fe_{0.34}Mg_{0.62}Al_{0.28}Si_{0.81}O_3$)	63:37	2200.39	0.26	After EC Exp	313(1)

Table S3. Best-fit hyperfine parameters of the Mössbauer spectra.

Run no.		Fe^{2+}				Fe^{3+}				χ^2
		CS(mm/s)	FWHM(mm/s)	QS(mm/s)	Areas(%)	CS(mm/s)	FWHM(mm/s)	QS(mm/s)	Areas(%)	
S8059	Before EC	1.11(2)	1.14(3)	2.12(4)	62.46	0.29(2)	0.97(7)	1.30(4)	37.55	2.82
	After EC	1.14(2)	1.11(4)	2.08(6)	65.97	0.36(2)	0.75(7)	1.17(5)	34.03	0.99
I1755	Before EC	1.19(2)	0.89(7)	1.81(3)	54.27	0.44(1)	0.59(4)	0.99(3)	45.73	1.11
	After EC	1.17(4)	0.97(11)	1.91(5)	64.97	0.45(2)	0.59(4)	0.87(6)	35.03	1.13
I1760	Before EC	1.19(3)	1.06(9)	1.74(4)	53.40	0.48(2)	0.61(4)	0.91(4)	46.60	1.07
	After EC	1.14(2)	0.97(6)	1.87(3)	59.46	0.45(2)	0.67(7)	0.95(6)	40.54	1.06
I1764	Before EC	1.16(20	1.03(6)	1.82(3)	35.52	0.43(1)	0.57(2)	0.94(1)	64.48	1.24
	After EC	1.16(3)	1.05(9)	1.84(4)	35.44	0.46(1)	0.58(2)	0.92(2)	64.56	1.01
I1767	Before EC	1.16(1)	1.06(4)	1.79(2)	40.59	0.44(1)	0.58(1)	0.92(1)	59.41	1.60
	After EC	1.19(2)	1.02(6)	1.84(2)	52.77	0.46(1)	0.59(3)	0.97(2)	47.23	1.12
I1779	Before EC	1.12(2)	1.03(4)	1.82(2)	35.83	0.45(1)	0.54(2)	0.90(1)	64.17	1.49
	After EC	1.12(3)	0.92(6)	2.09(5)	63.48	0.47(3)	0.71(6)	1.01(8)	36.52	1.09

Numbers in parentheses are the errors by fitting of Mossbauer spectra

REFERENCES

1. W. F. McDonough, S.-s. Sun, The composition of the Earth. *Chem. Geol.* **120**, 223–253 (1995).

2. S. M. Dorfman, T. S. Duffy, Effect of Fe-enrichment on seismic properties of perovskite and post-perovskite in the deep lower mantle. *Geophys. J. Int.* **197**, 910–919 (2014).

3. M. D. Ballmer, C. Houser, J. W. Hernlund, R. M. Wentzcovitch, K. Hirose, Persistence of strong silica-enriched domains in the Earth's lower mantle. *Nat. Geosci.* **10**, 236–240 (2017).

4. D. J. Frost, C. A. McCammon, The redox state of Earth's mantle. *Annu. Rev. Earth Planet. Sci.* **36**, 389–420 (2008).

5. S. Kadoya, D. C. Catling, R. W. Nicklas, I. S. Puchtel, A. D. Anbar, Mantle data imply a decline of oxidizable volcanic gases could have triggered the Great Oxidation. *Nat. Commun.* **11**, 2774 (2020).

6. Q. Yuan, M. Li, S. J. Desch, B. Ko, H. Deng, E. J. Garnero, T. S. J. Gabriel, J. A. Kegerreis, Y. Miyazaki, V. Eke, P. D. Asimow, Moon-forming impactor as a source of Earth's basal mantle anomalies. *Nature* **623**, 95–99 (2023).

7. E. J. Garnero, A. K. McNamara, S.-H. Shim, Continent-sized anomalous zones with low seismic velocity at the base of Earth's mantle. *Nat. Geosci.* **9**, 481–489 (2016).

8. F. V. Kaminsky, J.-F. Lin, Iron partitioning in natural lower-mantle minerals: Toward a chemically heterogeneous lower mantle. *Am. Mineral.* **102**, 824–832 (2017).

9. T. Irifune, T. Tsuchiya, "Mineralogy of the Earth-phase transitions and mineralogy of the lower mantle," in *Mineral Physics* (Elsevier, 2007), pp. 33–62.

10. I. Mosca, L. Cobden, A. Deuss, J. Ritsema, J. Trampert, Seismic and mineralogical structures of the lower mantle from probabilistic tomography. *J. Geophys. Res. Solid Earth* **117**, B06304 (2012).

11. T. Ishii, H. Kojitani, M. Akaogi, Phase relations of harzburgite and MORB up to the uppermost lower mantle conditions: Precise comparison with pyrolite by multisample cell

high-pressure experiments with implication to dynamics of subducted slabs. *J. Geophys. Res. Solid Earth* **124**, 3491–3507 (2019).

12. T. Ishii, N. Miyajima, G. Criniti, Q. Hu, K. Glazyrin, T. Katsura, High pressure-temperature phase relations of basaltic crust up to mid-mantle conditions. *Earth Planet. Sci. Lett.* **584**, 117472 (2022).

13. T. Yoshino, T. Katsura, Effect of iron content on electrical conductivity of ringwoodite, with implications for electrical structure in the transition zone. *Phys. Earth Planet. Inter.* **174**, 3–9 (2009).

14. L. Dai, S. Karato, The effect of pressure on the electrical conductivity of olivine under the hydrogen-rich conditions. *Phys. Earth Planet. Inter.* **232**, 51–56 (2014).

15. N. Purevjav, N. Tomioka, S. Yamashita, K. Shinoda, S. Kobayashi, K. Shimizu, M. Ito, S. Fu, J. Gu, C. Hoffmann, J.-F. Lin, T. Okuchi, Hydrogen incorporation mechanism in the lower-mantle bridgmanite. *Am. Mineral.* **109**, 1036–1044 (2024).

16. J.-P. Poirier, J. Peyronneau, "Experimental determination of the electrical conductivity of the material of the Earth's lower mantle," in *High-Pressure Research: Application to Earth and Planetary Sciences* (American Geophysical Union, 2013), pp. 77–87.

17. X. Li, R. Jeanloz, Effect of iron content on the electrical conductivity of perovskite and magnesiowüstite assemblages at lower mantle conditions. *J. Geophys. Res. Solid Earth* **96**, 6113–6120 (1991).

18. J. Sun, A. Kelbert, G. D. Egbert, Ionospheric current source modeling and global geomagnetic induction using ground geomagnetic observatory data. *J. Geophys. Res. Solid Earth* **120**, 6771–6796 (2015).

19. F. Deschamps, A. Khan, Electrical conductivity as a constraint on lower mantle thermo-chemical structure. *Earth Planet. Sci. Lett.* **450**, 108–119 (2016).

20. R. Sinmyo, G. Pesce, E. Greenberg, C. McCammon, L. Dubrovinsky, Lower mantle electrical conductivity based on measurements of Al, Fe-bearing perovskite under lower mantle conditions. *Earth Planet. Sci. Lett.* **393**, 165–172 (2014).

21. Y. Xu, C. McCammon, Evidence for ionic conductivity in lower mantle $(Mg,Fe)(Si,Al)O_3$ perovskite. *J. Geophys. Res. Solid Earth* **107**, 1–7 (2002).

22. Y. Xu, C. McCammon, B. T. Poe, The effect of alumina on the electrical conductivity of silicate perovskite. *Science* **282**, 922–924 (1998).

23. J. Peyronneau, J. P. Poirier, Electrical conductivity of the Earth's lower mantle. *Nature* **342**, 537–539 (1989).

24. T. Katsura, A revised adiabatic temperature profile for the mantle. *J. Geophys. Res. Solid Earth* **127**, e2021JB023562 (2022).

25. T. Yoshino, S. Kamada, C. Zhao, E. Ohtani, N. Hirao, Electrical conductivity model of Al-bearing bridgmanite with implications for the electrical structure of the Earth's lower mantle. *Earth Planet. Sci. Lett.* **434**, 208–219 (2016).

26. L. Waszek, B. Tauzin, N. C. Schmerr, M. D. Ballmer, J. C. Afonso, A poorly mixed mantle transition zone and its thermal state inferred from seismic waves. *Nat. Geosci.* **14**, 949–955 (2021).

27. T. J. Shankland, J. Peyronneau, J.-P. Poirier, Electrical conductivity of the Earth's lower mantle. *Nature* **366**, 453–455 (1993).

28. X. Huang, Y. Xu, S. Karato, Water content in the transition zone from electrical conductivity of wadsleyite and ringwoodite. *Nature* **434**, 746–749 (2005).

29. L. M. Hirsch, T. J. Shankland, A. G. Duba, Electrical conduction and polaron mobility in Fe-bearing olivine. *Geophys. J. Int.* **114**, 36–44 (1993).

30. T. Yoshino, A. Shimojuku, S. Shan, X. Guo, D. Yamazaki, E. Ito, Y. Higo, K. Funakoshi, Effect of temperature, pressure and iron content on the electrical conductivity of olivine and its high-pressure polymorphs. *J. Geophys. Res. Solid Earth* **117**, 2011JB008774 (2012).

31. T. Yoshino, D. Yamazaki, E. Ito, T. Katsura, No interconnection of ferro-periclase in post-spinel phase inferred from conductivity measurement. *Geophys. Res. Lett.* **35**, 2008GL035932 (2008).

32. L. Stixrude, C. Lithgow-Bertelloni, Thermodynamics of mantle minerals—III: The role of iron. *Geophys. J. Int.* **237**, 1699–1733 (2024).

33. R. Huang, T. Boffa Ballaran, C. A. McCammon, N. Miyajima, D. Dolejš, D. J. Frost, The composition and redox state of bridgmanite in the lower mantle as a function of oxygen fugacity. *Geochim. Cosmochim. Acta* **303**, 110–136 (2021).

34. V. Potapkin, C. McCammon, K. Glazyrin, A. Kantor, I. Kupenko, C. Prescher, R. Sinmyo, G. V. Smirnov, A. I. Chumakov, R. Rüffer, L. Dubrovinsky, Effect of iron oxidation state on the electrical conductivity of the Earth's lower mantle. *Nat. Commun.* **4**, 1427 (2013).

35. A. V. Grayver, F. D. Munch, A. V. Kuvshinov, A. Khan, T. J. Sabaka, L. Tøffner-Clausen, Joint inversion of satellite-detected tidal and magnetospheric signals constrains electrical conductivity and water content of the upper mantle and transition zone. *Geophys. Res. Lett.* **44**, 6074–6081 (2017).

36. A. Kuvshinov, A. Grayver, L. Tøffner-Clausen, N. Olsen, Probing 3-D electrical conductivity of the mantle using 6 years of Swarm, CryoSat-2 and observatory magnetic data and exploiting matrix Q-responses approach. *Earth Planets Space* **73**, 67 (2021).

37. F. Civet, E. Thébault, O. Verhoeven, B. Langlais, D. Saturnino, Electrical conductivity of the Earth's mantle from the first Swarm magnetic field measurements. *Geophys. Res. Lett.* **42**, 3338–3346 (2015).

38. C. Püthe, A. Kuvshinov, A. Khan, N. Olsen, A new model of Earth's radial conductivity structure derived from over 10 yr of satellite and observatory magnetic data. *Geophys. J. Int.* **203**, 1864–1872 (2015).

39. A. Kuvshinov, N. Olsen, A global model of mantle conductivity derived from 5 years of CHAMP, Ørsted, and SAC-C magnetic data. *Geophys. Res. Lett.* **33**, 2006GL027083 (2006).

40. S. Ozaydin, L. Li, U. Singh, P. F. Rey, M. C. Manassero, pide: Petrophysical Interpretation tools for geoDynamic Exploration. *J. Open Source Softw.* **10**, 7021 (2025).

41. A. Semenov, A. Kuvshinov, Global 3-D imaging of mantle conductivity based on inversion of observatory C-responses—II. Data analysis and results. *Geophys. J. Int.* **191**, 965–992 (2012).

42. S. Cottaar, V. Lekic, Morphology of seismically slow lower-mantle structures. *Geophys. J. Int.* **207**, 1122–1136 (2016).

43. B. H. Heyn, C. P. Conrad, R. G. Trønnes, How thermochemical piles can (periodically) generate plumes at their edges. *J. Geophys. Res. Solid Earth* **125**, e2019JB018726 (2020).

44. C. Zhao, E. J. Garnero, A. K. McNamara, N. Schmerr, R. W. Carlson, Seismic evidence for a chemically distinct thermochemical reservoir in Earth's deep mantle beneath Hawaii. *Earth Planet. Sci. Lett.* **426**, 143–153 (2015).

45. S. Spasojevic, M. Gurnis, R. Sutherland, Mantle upwellings above slab graveyards linked to the global geoid lows. *Nat. Geosci.* **3**, 435–438 (2010).

46. X. Liu, S. Zhong, Constraining mantle viscosity structure for a thermochemical mantle using the geoid observation. *Geochem. Geophys. Geosystems* **17**, 895–913 (2016).

47. A. W. Hofmann, Chemical differentiation of the Earth: The relationship between mantle, continental crust, and oceanic crust. *Earth Planet. Sci. Lett.* **90**, 297–314 (1988).

48. J. Immoor, L. Miyagi, H.-P. Liermann, S. Speziale, K. Schulze, J. Buchen, A. Kurnosov, H. Marquardt, Weak cubic $CaSiO_3$ perovskite in the Earth's mantle. *Nature* **603**, 276–279 (2022).

49. B. Tauzin, L. Waszek, M. D. Ballmer, J. C. Afonso, T. Bodin, Basaltic reservoirs in the Earth's mantle transition zone. *Proc. Natl. Acad. Sci. U.S.A.* **119**, e2209399119 (2022).

50. S. Kaneshima, Seismic scatterers in the lower mantle near subduction zones. *Geophys. J. Int.* **219**, S2–S20 (2019).

51. D. L. Lakshtanov, S. V. Sinogeikin, K. D. Litasov, V. B. Prakapenka, H. Hellwig, J. Wang, C. Sanches-Valle, J.-P. Perrillat, B. Chen, M. Somayazulu, J. Li, E. Ohtani, J. D. Bass, The post-stishovite phase transition in hydrous alumina-bearing SiO_2 in the lower mantle of the earth. *Proc. Natl. Acad. Sci. U.S.A.* **104**, 13588–13590 (2007).

52. E. Ohtani, T. Ishii, Role of water in dynamics of slabs and surrounding mantle. *Prog Earth Planet Sci* **11**, 65 (2024).

53. A. Grayver, Unravelling the electrical conductivity of earth and planets. *Surv. Geophys.* **45**, 187–238 (2024).

54. J. Velímský, O. Knopp, Lateral variations of electrical conductivity in the lower mantle constrained by Swarm and CryoSat-2 missions. *Earth Planets Space* **73**, 4 (2021).

55. C. Prescher, C. McCammon, L. Dubrovinsky, MossA: A program for analyzing energy-domain Mössbauer spectra from conventional and synchrotron sources. *J. Appl. Cryst.* **45**, 329–331 (2012).

56. C. McCammon, Perovskite as a possible sink for ferric iron in the lower mantle. *Nature* **387**, 694–696 (1997).

57. D. P. Dobson, J. P. Brodholt, The electrical conductivity of the lower mantle phase magnesiowüstite at high temperatures and pressures. *J. Geophys. Res. Solid Earth* **105**, 531–538 (2000).

58. H. Fei, R. Huang, X. Yang, $CaSiO_3$ perovskite may cause electrical conductivity jump in the topmost lower mantle. *Geophys. Res. Lett.* **44**, 10226–10232 (2017).

59. K. D. Litasov, H. Kagi, A. Shatskiy, E. Ohtani, D. L. Lakshtanov, J. D. Bass, E. Ito, High hydrogen solubility in Al-rich stishovite and water transport in the lower mantle. *Earth Planet. Sci. Lett.* **262**, 620–634 (2007).

60. T. Yoshino, A. Shimojuku, D. Li, Electrical conductivity of stishovite as a function of water content. *Phys. Earth Planet. Inter.* **227**, 48–54 (2014).

61. K. Ohta, K. Hirose, M. Ichiki, K. Shimizu, N. Sata, Y. Ohishi, Electrical conductivities of pyrolitic mantle and MORB materials up to the lowermost mantle conditions. *Earth Planet. Sci. Lett.* **289**, 497–502 (2010).

62. K. Han, S. M. Clark, Review of calculating the electrical conductivity of mineral aggregates from constituent conductivities. *Solid Earth Sci.* **6**, 111–128 (2021).

63. P. Tarits, M. Mandéa, The heterogeneous electrical conductivity structure of the lower mantle. *Phys. Earth Planet. Inter.* **183**, 115–125 (2010).

64. A. Kelbert, A. Schultz, G. Egbert, Global electromagnetic induction constraints on transition-zone water content variations. *Nature* **460**, 1003–1006 (2009).

65. T. Katsura, K. Sato, E. Ito, Electrical conductivity of silicate perovskite at lower-mantle conditions. *Nature* **395**, 493–495 (1998).

66. F. D. Munch, B. Romanowicz, S. Mukhopadhyay, M. L. Rudolph, Deep mantle plumes feeding periodic alignments of asthenospheric fingers beneath the central and southern Atlantic Ocean. *Proc. Natl. Acad. Sci. U.S.A.* **121**, e2407543121 (2024).

67. Y. Fukao, M. Obayashi, Subducted slabs stagnant above, penetrating through, and trapped below the 660 km discontinuity. *J. Geophys. Res. Solid Earth* **118**, 5920–5938 (2013).
